# Frost tolerance improvement in pea and white lupin by a high-throughput phenotyping platform

**DOI:** 10.3389/fpls.2024.1490577

**Published:** 2024-12-20

**Authors:** Nicolò Franguelli, Daniele Cavalli, Tommaso Notario, Luciano Pecetti, Paolo Annicchiarico

**Affiliations:** Council for Agricultural Research and Economics (CREA), Research Centre for Animal Production and Aquaculture, Lodi, Italy

**Keywords:** abiotic stress, cold tolerance, cool-season grain legumes, low temperature stress, winter mortality, winter plant survival, *Pisum sativum*, *Lupinus albus*

## Abstract

The changing climate could expand northwards in Europe the autumn sowing of cool-season grain legumes to take advantage of milder winters and to escape the increasing risk of terminal drought. Greater frost tolerance is a key breeding target because sudden frosts following mild-temperature periods may produce high winter mortality of insufficiently acclimated plants. The increasing year-to-year climate variation hinders the field-based selection for frost tolerance. This study focused on pea and white lupin with the objectives of (i) optimizing an easy-to-build, high-throughput phenotyping platform for frost tolerance assessment with respect to optimal freezing temperatures, and (ii) verifying the consistency of genotype plant mortality responses across platform and field conditions. The platform was a 13.6 m^2^ freezing chamber with programmable temperature in the range of −15°C to 25°C. The study included 11 genotypes per species with substantial variation for field-based winter plant survival. Plant seedlings were evaluated under four freezing temperature treatments, i.e., −7°C, −9°C, −11°C, and −13°C, after a 15-day acclimation period at 4°C. Genotype plant mortality and lethal temperature corresponding to 50% mortality (LT_50_) were assessed at the end of a regrowth period, whereas biomass injury was observed through a 10-level visual score based on the amount of necrosis and mortality after recovery and regrowth. On average, pea displayed higher frost tolerance than white lupin (mean LT_50_ of −12.8 *versus* −11.0°C). The genotype LT_50_ values ranged from −11.6°C to −14.5°C for pea and from −10.0°C to −12.0°C for lupin. The freezing temperature that maximized the genotype mortality variation was −13°C for pea and −11°C for lupin. The genotype mortality at these temperatures exhibited high correlations with LT_50_ values (0.91 for pea and 0.94 for lupin) and the biomass injury score (0.98 for pea and 0.97 for lupin). The frost tolerance responses in the platform showed a good consistency with the field-based winter survival of the genotypes. Our study indicates the reliability of genotype frost tolerance assessment under artificial conditions for two cool-season grain legumes, offering a platform that could be valuable for crop improvement as well as for genomics and ecophysiological research.

## 1 Introduction

Legume cultivation has a highly positive impact on agri-food systems by increasing the availability of biologically fixed nitrogen, enhancing soil quality, promoting biodiversity, and mitigating the impact of weeds and pests (Karkanis et al., 2016; Nemecek et al., 2008). For European agriculture, greater legume cultivation would help reduce its significant deficit and reliance on imported high-protein feedstuff (which contributes to Amazon deforestation: Boerema et al., 2016) and meet the increasing industry demand for novel protein-rich foods (Lucas et al., 2015; van Loon et al., 2023). Pea (*Pisum sativum* L.) and white lupin (*Lupinus albus* L.) are promising cool-season grain legume crops for southern Europe. Compared to other pulses, pea has a higher yield and energy production, while lupin maximizes protein yield per unit area due to its outstanding seed protein content (Karkanis et al., 2016; Annicchiarico, 2008; Cernay et al., 2016). However, greater plant breeding effort is indispensable to reduce the yield gap with cereals and increase the economic sustainability of these crops (Rubiales et al., 2021).

Cool-season grain legumes are typically sown in autumn in mild-winter regions and in late winter or early spring in cold-prone regions of Europe. The changing climate is expected to expand the autumn sowing the northwards, allowing crops to benefit from milder winters and escape the increasing risk of terminal drought through earlier crop maturity (Alessandri et al., 2014). Crop frost tolerance is a key breeding target in this context, not only to withstand low temperature stress in cold regions but also because sudden frost events following mild-temperature periods may produce high winter plant mortality due to insufficient cold acclimation (Annicchiarico and Iannucci, 2007). Various stresses may concurrently affect winter survival, including frost, waterlogging, and fungal pathogens (Bélanger et al., 2006). However, frost has prominent importance and can be faced by plants through frost avoidance and frost tolerance mechanisms (Janská et al., 2010). Frost avoidance is based on delayed flowering (aimed to protect the very sensitive reproductive organs) (Maqbool et al., 2010; Siddique et al., 1999), which is primarily achieved through greater vernalization requirements in white lupin (Huyghe and Papineau, 1990) and by photoperiodic control and/or high growing degree days requirements in pea (Summerfield and Roberts, 1988; Lejeune-Hénaut et al., 1999; Weller et al., 1997). In target regions possibly subjected to both low winter temperatures and terminal drought, late flowering and crop maturity may ensure frost avoidance and a higher yield in cold, relatively moisture-favorable years while being associated with greater drought susceptibility and lower yield in relatively mild, drought-prone years (Annicchiarico et al., 2011, 2019). For plant breeders, this dilemma can be coped with by selecting materials with intermediate flowering times but intrinsic frost and drought tolerance. Intrinsic drought tolerance could be expressed by a positive deviation from the genotype yield expected according to its onset of flowering (Annicchiarico et al., 2017; Pecetti et al., 2023). Intrinsic frost tolerance of cold-acclimated plants could likewise be expressed by a positive deviation from the genotype winter plant survival expected according to its onset of flowering. The frost tolerance mechanisms of cool-season grain legumes are based on physiological modifications to prevent or resist intracellular ice formation (Bourion et al., 2003), such as decreased shoot water content during cold acclimation (Sallam et al., 2015), increased cell membrane stability through changes in the lipid-to-protein ratio and the membrane lipid unsaturation level (Arbaoui and Link, 2008), and accumulation of osmoprotectant compounds such as proline, glycine betaine, mannitol, sucrose, raffinose, stachyose, and specific proteins that protect against dehydration (Link et al., 2010).

Breeding for improved frost tolerance under field conditions is complicated by the wide and increasing climate variation across years, which reduces the applicability, efficiency, and replicability of the selection (Avia et al., 2013). A reliable assessment of frost tolerance in controlled environments could overcome these limitations and allow, in addition, for off-season selections (Stoddard et al., 2006). Its assessment on seedlings rules out any effect of flowering time and focuses, therefore, on intrinsic frost tolerance. The assessment requires a period of cold acclimation (hardening) above 0°C temperature, the duration of which increases the frost tolerance of relatively winter-hardy material (Swensen and Murray, 1983). Hardening was performed at 4°C over 2 to 4 weeks in most freezing tolerance studies on pea (Auld et al., 1983; Murray et al., 1988; Meyer and Badaruddin, 2001; Homer et al., 2016). Subsequently, slow cooling toward the stress temperature is essential to ensure sufficient time for water redistribution, with a cooling rate not exceeding 2°C/h (Murray et al., 1988). An accurate assessment of mortality can only be made after a minimum recovery period of 3 weeks under favorable temperatures (Murray et al., 1988). Besides plant mortality, genotype frost tolerance could also be expressed by a visual score based on the amount of necrotic areas and other traits (Ali et al., 2016; Beji et al., 2020). The assessment of the genotype lethal temperature 50 (LT_50_), i.e., the freezing temperature corresponding to 50% of mortality, requires the evaluation of plant mortality across a set of freezing temperatures and may, therefore, be operationally less adequate than the evaluation of plant mortality at just one optimal freezing temperature when assessing frost tolerance in large numbers of genotypes as in selection trials (Waalen et al., 2011). Such optimal temperature should ensure the maximization of genotype variation for plant mortality, and may approach the genotype mean value of LT_50_ in studies including a sample of genotypes representative of the crop frost tolerance variation. Various studies suggested that this temperature may fall in the range of −7°C to −9°C (Auld et al., 1983; Swensen and Murray, 1983; Murray et al., 1988; Cousin et al., 1993; Homer et al., 2016) for pea based on small sets of genotypes mostly selected several decades ago, whereas no information is available for white lupin. For pea, an official frost tolerance evaluation test prescribes the assessment of candidate varieties at −8°C freezing temperature (Wery et al., 1994).

We recently established an easy-to-build, high-throughput phenotyping platform for frost tolerance assessment represented by a 13.6 m^2^ growth freezing chamber with programmable temperature to be used for the selection and genomic prediction of frost tolerance in cool-season grain legumes. This study assessed plant mortality, LT_50_ values, and the biomass injury visual score of 11 genotypes of pea and 11 of white lupin encompassing a wide range of winter mortality in earlier field trials in northern Italy, with the objectives of (i) optimizing the frost tolerance platform with respect to optimal freezing temperatures for each species and (ii) verifying the consistency of genotype plant mortality responses across platform and field conditions.

## 2 Materials and methods

### 2.1 Plant material

The experiment included 11 genotypes of pea and 11 of white lupin comprising commercial cultivars, landraces, and breeding lines, which were selected within each species to represent a wide variation in winter survival based on the results of previous field trials in northern Italy (**Table 1**). Based on winter plant mortality observed under field conditions in separate earlier experiments, we classified the genotypes into three broad classes of winter hardiness: high, intermediate, and low. One pea genotype, namely, the French landrace Champagne, was selected as a standard of extreme field-based winter hardiness according to Prieur and Cousin (1978) and Dumont et al. (2011).

**Table 1:** Name, origin (for cultivars or landraces), plant winter mortality in autumn-sown field experiments in northern Italy, and suggested winter hardiness based on these experiments for 11 pea and 11 white lupin genotypes.

Genotype name [alias] (origin)	Mortality (%)^a^	Winter hardiness
Pea breeding lines and parent lines^b^		
KI_L38	3.3	High
KA_37	21.3	High
KI_118	67.0	Low
KA_19	86.0	Low
Isard	10.1	High
Kaspa	51.0	Intermediate
LSD (*P* < 0.05)	16.0	
Pea commercial cultivars^c^		
Dolmen (France)	2.6	High
Dove (France)	6.8	Intermediate
Kaspa (Australia)	7.8	Intermediate
Guifilo (Spain)	9.5	Intermediate
Catania (France)	24.7	Low
LSD (*P* < 0.05)	7.4	
Pea landraces		
Champagne (France)^d^	−	High
Lupin breeding lines^e^		
PLI4-3	16.1	High
PLI7-50 [Arsenio]	37.3	Intermediate
PLI-P3	88.8	Low
LSD (*P* < 0.05)	19.3	
Lupin commercial cultivars and landraces^f^		
Ludet (France)	0.0	High
Adam (France)	5.9	High
Amiga (France)	81.6	Low
Calabria [LAP 0108] (Italy)	2.2	High
GR56 [LAP 0019] (Greece)	8.6	High
E80 [LAP 0041] (Portugal)	33.6	Intermediate
Egypte11 [LAP 0086] (Egypt)	97.9	Low
LA 559 [LAP 0079] (Ethiopia)	93.0	Low
LSD (*P* < 0.05)	12.8	

a LSD relative to the values of subsets of genotypes as identified by the sub-headings in the first column.

b One environment with an absolute minimum temperature of −11.6°C [see Annicchiarico et al. (2019)].

c Average of two autumn sowing dates in one environment with an absolute minimum temperature of −7.8°C [see Annicchiarico and Iannucci (2007)].

d Described as highly winter-hardy by Prieur and Cousin (1978) and by Dumont et al. (2011).

e One environment with an absolute minimum temperature of −13.5°C (Lodi, cropping season 2005–2006) (Annicchiarico, unpublished data).

f One environment with an absolute minimum temperature of −9.0°C [see Annicchiarico et al. (2010)].

### 2.2 Frost tolerance evaluation experiment

The phenotyping platform consisted of a freezing chamber 4.80 m long × 2.84 m wide × 2.46 m high, with programmable temperature in the range of −15°C to 25°C. The chamber was equipped with eight Combo 300-W (C-LED, Bologna) lamps arranged in two rows, placed at a height of 1.6 m from the floor and about 0.9 m above the plant material. Individual test plants were sown at a depth of 2.5 cm into polystyrene plug trays composed of cells measuring 5 cm × 5 cm and 15 cm in depth filled with a commercial growing substrate that included peat corrected for acidity (pH = 6.0) and mineral compound fertilizer NPK (substrate SER CA-V7, Vigorplant, Piacenza, Italy). The plants were placed side by side on four large trolleys fitting into the chamber. Each experimental unit included a set of 10 adjacent plants.

The frost tolerance of the 22 genotypes was tested under four freezing treatments: −7°C, −9°C, −11°C, and −13°C. Plant acclimation took place at 4°C over 15 days, a shorter duration than in most of the earlier pea freezing tolerance studies but consistent with the trend toward milder winters and reduced hardening periods in agricultural environments caused by the changing climate.

The experiment included four experimental units (organized in blocks) per genotype and treatment. Within each treatment, the genotypes were arranged according to a group balanced block layout (Gomez and Gomez, 1984) holding species on main plots and the different genotypes of the two species on subplots. Operationally, the four blocks were subdivided into two growth cycles of two blocks each, which were performed sequentially using exactly the same protocol.

The seeds were pre-germinated on filter paper in Petri dishes for approximately 48 h at 19°C before being transplanted into the plug trays. The evaluation protocol included (i) 10 days of growth at 22.5°C with 12 h of daylight, (ii) 15 days of cold acclimation (hardening) at 4°C with 10 h of daylight, (iii) 12 h of cooling at −3°C in the dark, (iv) 4 h of freezing treatment, (v) 6 days of recovery at 4°C with 10 h of daylight, and (vi) 15 days of regrowth at 15/20°C (night/day) with 12 h of daylight (**Supplementary Table S1**). The plants were irrigated every 2 days during growth, recovery, and regrowth, while irrigation was suspended from the beginning of hardening to the end of the freezing treatment. The decrease in temperature toward the freezing point and the subsequent increase in temperature occurred at a rate of 1°C/h, according to a pattern described in **Supplementary Figure S1** for one test temperature. Air and soil temperatures were monitored with two Tinytag Plus 2 TGP-4510 (Gemini, Chichester) dataloggers to ensure compliance with the protocol.

### 2.3 Data collection

Frost tolerance was assessed using two criteria: plant mortality (i.e., the number of dead plants/total number of plants after hardening), and the level of injury to the aerial biomass measured through a visual score on individual plants and then averaged over plants of the experimental unit. The biomass injury visual score comprised the following 10 levels of increasing damage, which were based on observations at the end of the recovery period to evaluate mild injuries and at the end of regrowth to assess severe damage, such as mortality (Murray et al., 1988): (1) no visible damage, (2) loss of leaf turgidity for lupin and presence of dried tendrils for pea, (3) presence of dotted necrosis for lupin and leaf yellowing for pea, (4) presence of few necrotic spots, (5) up to 50% of leaf biomass necrotized, (6) between 50% and 90% of leaf biomass necrotized, (7) almost 100% of leaf biomass necrotized, (8) all of the biomass necrotized but a new shoot has started to grow, (9) the plant is severely damaged, with a very high expected probability of death, and (10) the plant is dead. For mortality assessment, plants that scored 9 and 10 were considered dead.

### 2.4 Statistical analysis

To compute the LT_50_ values, we fitted the following generalized linear model with the probit link function *ϕ* ^–1^:


$$\;\phi^{-1}\left[E\left(m_{g,b}\right)\right]=\beta_{g}T+\nu_{g}+\alpha_{b}$$

In the equation, the expectations of plant mortality ratios $E\left(m_{g,b}\right)$ are binomially distributed and depend on the fixed effects of genotype $g^{th}$ and block $b^{th}$ , as well as on the frost treatment temperature *T*, expressed as a covariate, with the slope $\beta_{g}$ depending on the genotype. The significance of each factor was assessed via a likelihood ratio test. Two standard model control techniques were applied to test the reliability of the model: a graphical assessment of raw residuals and Pearson’s residuals against fitted values, and the test of homogeneity of the means. The LT_50_ values were computed for each genotype within block according to the procedure described in Lei and Sun (2018), namely, as the opposite of the ratio between the intercept ( $\nu_{g}+\alpha_{b}$ ) and the angular coefficient $(\beta_{g}$ ) of the model.

An analysis of variance, including the factors genotype and block, was performed separately for each species to detect significant differences among genotypes for (i) LT_50_, (ii) proportion of plant mortality following each freezing treatment, and (iii) biomass injury visual score following each freezing treatment. Mortality data were first transformed by using the arcsine square root transformation. We reported original data along with least significant difference (LSD) values back-transformed from LSD values obtained from the analysis of transformed data, and assessed the genotype differences by using Duncan’s test. The mean values of species for plant mortality and LT_50_ were compared according to the group balanced block lay-out, i.e., by testing the species factor on an error term represented by the species × block interaction. The consistency of genotype frost tolerance assessments based on LT_50_, plant mortality, and biomass injury score values was determined by using Pearson’s correlation analysis.

Statistical models were fitted by the *glm()* and *lm()* functions from the R-package “stats”. Duncan’s test was performed by using the *duncan.test()* function, and LSD values were computed by using the *LSD.test()* function from the R-package “agricolae” (Steel et al., 1997).

## 3 Results

**Figure 1:**
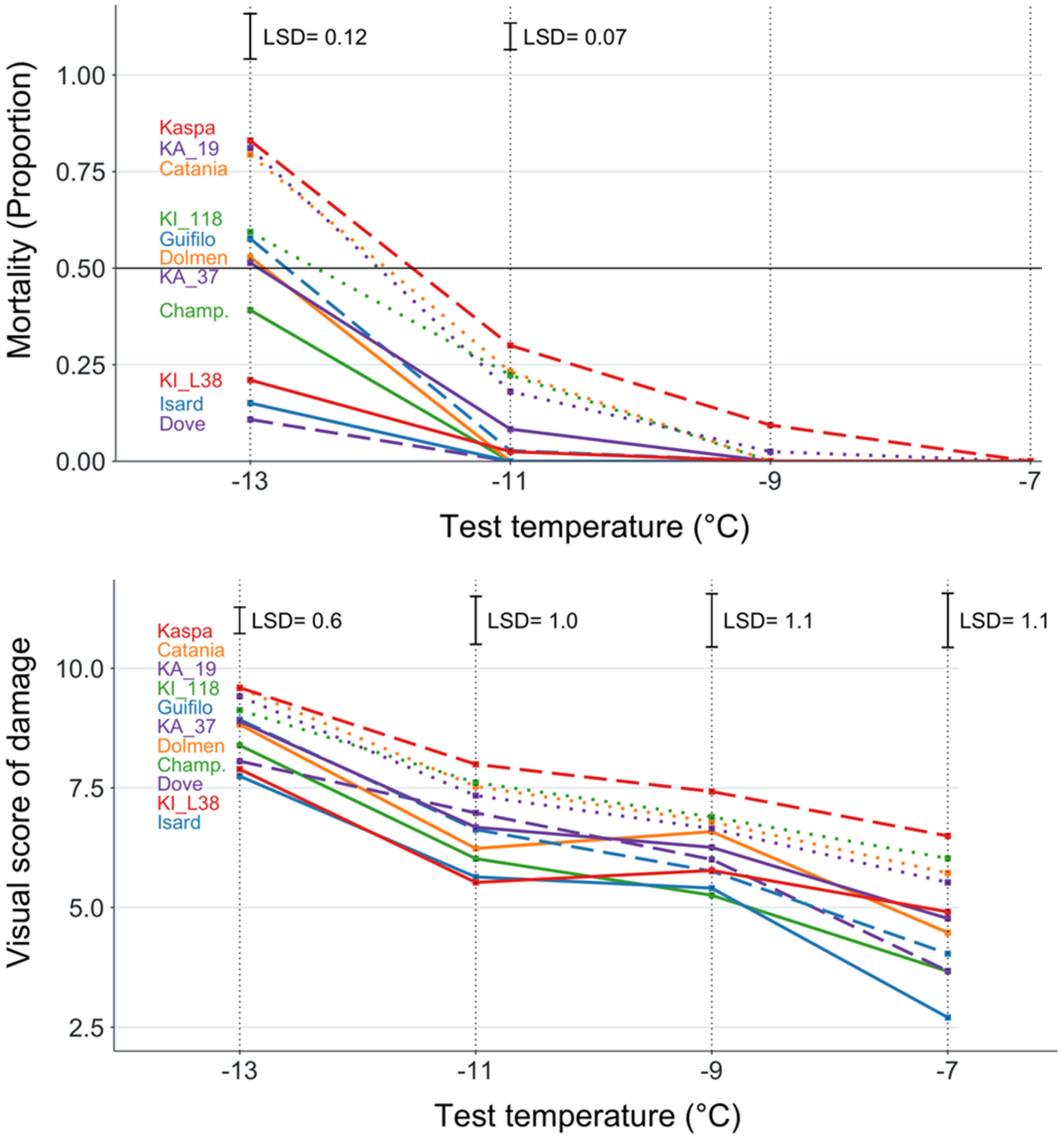
Plant mortality and biomass injury visual score for four freezing temperature treatments of 11 pea genotypes classified into three winter hardiness classes based on field-based winter mortality data (solid line, high winter hardiness; broken line, intermediate winter hardiness; dotted line, low winter hardiness). Least significant difference values at *P* < 0.05 reported only in the presence of overall genotype differences at *P* < 0.05.

On average, pea exhibited greater frost tolerance than white lupin as indicated by the lower LT_50_ (−12.8 *versus* −11.0°C; *P* < 0.01) and lower plant mortality at the lowest freezing temperature (0.50 *versus* 0.91; *P* < 0.01) in the analysis of variance-based species comparison. Within pea, the genotype values of LT_50_ ranged from −14.5°C for the breeding line KI_L38 to −11.6°C for the cultivar Kaspa (**Table 2**). The pea genotype plant mortality values showed no mortality at −7°C freezing temperature and did not differ significantly *(P* > 0.05) at −9°C (**Figure 1**). They displayed significant differences (*P* < 0.01) at lower temperatures and achieved the largest variation, ranging from 0.11 to 0.83, at −13°C (**Figure 1**, **Table 2**). The biomass injury visual score of the pea genotypes decreased with increasing freezing temperature but exhibited significant (*P* < 0.01) and similar extents of overall genotype variation across all freezing temperatures (**Figure 1**). The injury score values showed a high correlation (*r* ≥ 0.77, *P* < 0.01) across the four freezing temperatures (**Supplementary Table S2**). The high correlation (*P* < 0.001) of genotype mortality at −13°C with biomass injury score for the same temperature (*r* = 0.98) and LT_50_ (*r* = 0.91), and that between genotype values of the last two traits (*r* = 0.92), indicated a strong consistency between the main indicators of pea genotype frost tolerance.

**Table 2:** LT_50_ value, plant mortality proportion at the two lowest freezing temperatures, and biomass injury visual score (VS) after the highest and lowest freezing temperatures for 11 pea genotypes classified into three winter hardiness classes based on field-based winter mortality data.

Genotype	Winter hardiness	LT_50_ (°C)	Mortality, −11°C	Mortality, −13°C	VS, −7°C	VS, −13°C
KI_L38	High	−14.5 a	0.03 ab	0.21 abc	4.9 bcd	7.9 ab
Dove	Intermediate	−13.5 b	0.00 a	0.11 a	3.7 ab	8.1 ab
Isard	High	−13.4 b	0.00 a	0.15 ab	2.7 a	7.8 a
Champagne	High	−13.1 c	0.00 a	0.39 bcd	3.7 ab	8.4 bc
Dolmen	High	−13.0 cd	0.00 a	0.53 de	4.5 bc	8.8 cd
KA_37	High	−13.0 cd	0.08 abc	0.51 cd	4.8 bc	8.9 cd
Guifilo	Intermediate	−12.8 d	0.03 ab	0.58 de	4.0 b	8.9 cd
KI_118	Low	−12.4 e	0.22 cd	0.59 de	6.0 de	9.1 de
Catania	Low	−12.0 f	0.23 cd	0.79 e	5.7 cde	9.6 e
KA_19	Low	−11.9 f	0.18 bcd	0.81 e	5.5 cde	9.4 de
Kaspa	Intermediate	−11.6 g	0.30 d	0.83 e	6.5 e	9.6 e
LSD (*P* < 0.05)		0.3	0.07	0.12	1.1	0.6

Column means followed by different letter differs at *P* < 0.05 according to Duncan’s test.

**Table 2** reports the results of pea genotype comparisons relative to LT_50_, plant mortality at the two lowest freezing temperatures (those displaying a significant genotype variation), and the biomass injury score for the highest and lowest temperatures (i.e., the extreme temperature range). In general, the pea genotype mean separation was more sensitive for LT_50_ (where KI_L38 outperformed any other genotype at *P* < 0.05) than for the other traits (**Table 2**). All genotypes assigned to the high winter hardiness class on the basis of field observation exhibited low to fairly low values of LT_50_, plant mortality, and injury score, while all genotypes assigned to the low winter hardiness class showed fairly high to high values of these traits (**Table 2**). However, two genotypes in the intermediate winter hardiness class, namely, Dove and Kaspa, exhibited high and low frost tolerance, respectively, according to all traits. The highly winter-hardy genotype Champagne displayed high, but not outstanding, frost tolerance.

**Figure 2:**
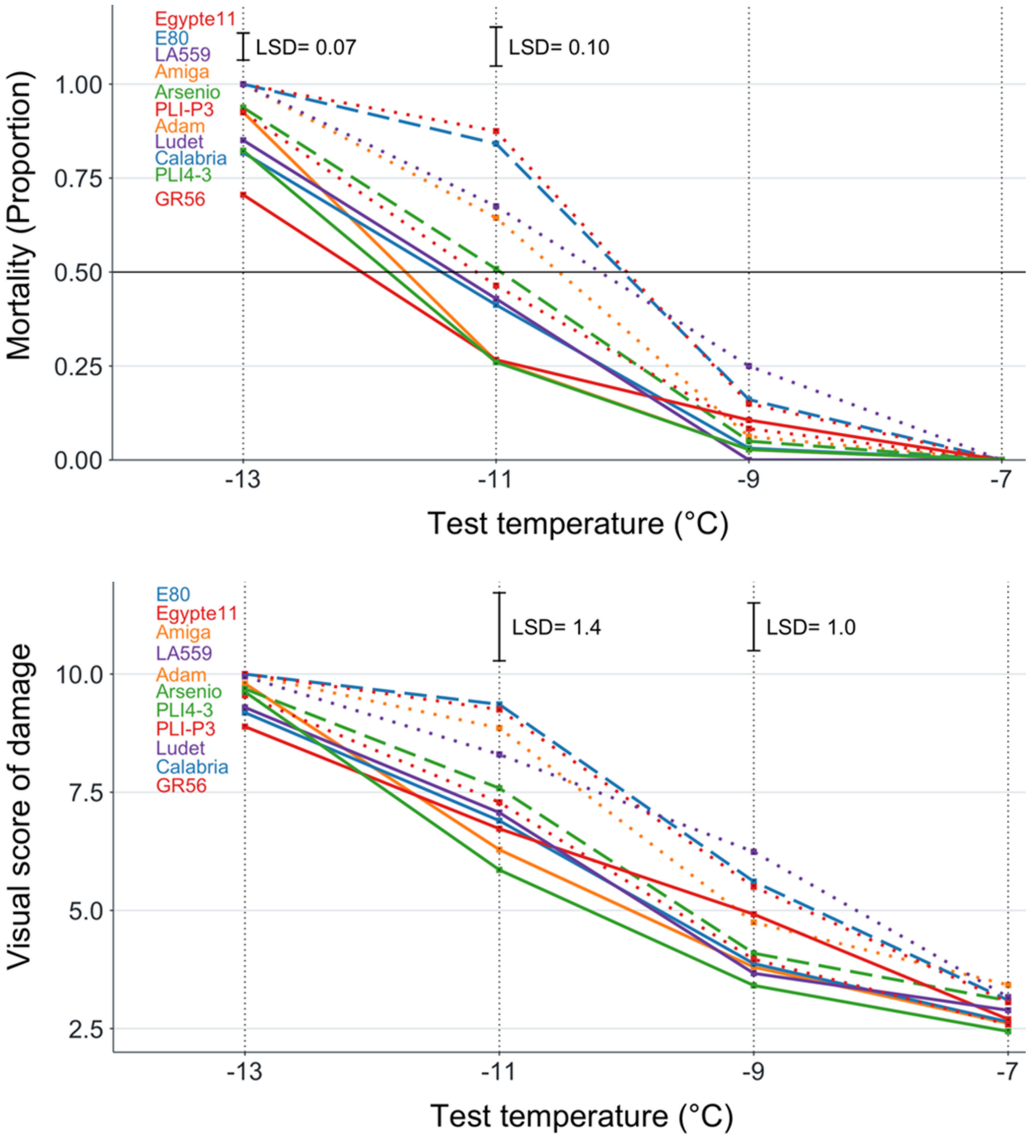
Plant mortality and biomass injury visual score for four freezing temperature treatments of 11 white lupin genotypes classified into three winter hardiness classes based on field-based winter mortality data (solid line, high winter hardiness; broken line, intermediate winter hardiness; dotted line, low winter hardiness). Least significant difference values at *P* < 0.05 reported only in the presence of overall genotype differences at *P* < 0.05.

The white lupin genotypes displayed LT_50_ values ranging from −12.0°C for the Greek landrace GR56 to −10.0°C for the Egyptian landrace Egypte11 and the Portuguese landrace E80. We observed no lupin genotype plant mortality at -7°C, and significant (*P* < 0.01) plant mortality variation only at −11°C and −13°C freezing temperatures (**Figure 2**). However, the widest variation for genotype mortality, in the range of 0.26 to 0.88, took place at −11°C in this species (**Figure 2**, **Table 3**). The variation for the biomass injury score achieved significance (*P* < 0.01) only for the two intermediate temperatures while being flattened toward low values at −7°C and high values at −13°C (**Figure 2**). A high consistency among major indicators of genotype frost tolerance was observed also in white lupin according to correlations (*P* < 0.001) of genotype mortality at −11°C with the biomass injury score for the same temperature (*r* = 0.97) and LT_50_ (*r* = 0.94), or that between values of the last two traits (*r* = 0.91). High correlations were also observed for other indicators of frost tolerance (**Supplementary Table S3**).

**Table 3:** LT_50_ value, plant mortality proportion, and biomass injury visual score (VS) for two freezing temperatures showing significant differences for 11 white lupin genotypes classified into three winter hardiness classes based on field-based winter mortality data.

Genotype	Winter hardiness	LT_50_ (°C)	Mortality, −11°C	Mortality, −13°C	VS, −9°C	VS, −11°C
GR56	High	−12.0 a	0.27 a	0.71 a	4.9 cd	6.7 abc
Calabria	High	−11.9 ab	0.41 ab	0.82 ab	3.9 abc	6.9 abc
PLI4-3	High	−11.8 b	0.26 a	0.82 ab	3.4 a	5.9 a
Adam	High	−11.5 c	0.26 a	0.93 bc	3.8 abc	6.3 ab
Ludet	High	−11.5 c	0.43 ab	0.85 abc	3.7 ab	7.1 abc
PLI-P3	Low	−11.1 d	0.46 ab	0.93 bc	4.0 abc	7.3 abc
Arsenio	Intermediate	−11.0 d	0.51 ab	0.94 bc	4.1 abc	7.6 bcd
Amiga	Low	−10.6 e	0.64 bc	1.00 c	4.7 bcd	8.9 de
LA559	Low	−10.1 f	0.68 bcd	1.00 c	6.2 e	8.3 cde
E80	Intermediate	−10.0 f	0.84 cd	1.00 c	5.6 de	9.4 e
Egypte11	Low	−10.0 f	0.88 d	1.00 c	5.5 de	9.3 e
LSD (*P* < 0.05)		0.2	0.10	0.07	1.0	1.4

Column means followed by different letters differ at *P* < 0.05 according to Duncan’s test.

The results for major indicators of frost tolerance reported for each white lupin genotype in **Table 3** indicated also for this species a good consistency between frost tolerance in the phenotyping platform and winter hardiness based on field observations. The five genotypes belonging to the high winter hardiness class were the top-ranking ones for frost tolerance according to LT_50_ values, plant mortality, or the biomass injury score for −11°C, whereas three genotypes in the low winter hardiness class out of four were bottom-ranking for all of these frost tolerance indicators. The only inconsistencies were represented by the genotype E80, which was susceptible to frost while belonging to the intermediate winter hardiness class, and the breeding line PLI-P3, which displayed intermediate frost tolerance while being assigned to the low winter hardiness class. In white lupin, too, LT_50_ exhibited more sensitive genotype mean separation than plant mortality or the injury score (**Table 3**).

## 4 Discussion

On average, pea exhibited greater frost tolerance than white lupin in this study. This result agrees with the greater average winter plant survival of pea relative to white lupin in a field-based assessment of a large number of varieties across climatically contrasting Italian environments (Annicchiarico and Iannucci, 2007). In general, however, pea is credited an intermediate winter hardiness among the cool-season grain legumes, being considered less winter-hardy than faba bean or lentil and more winter-hardy than chick pea (Murray et al., 1988).

Our results indicated a good consistency between major indicators of genotype frost tolerance observed in the phenotyping platform, namely, LT_50_, plant mortality at the freezing temperature that maximized the genotype variation, and biomass injury score for the same temperature or a slightly higher one. LT_50_ exhibited a more sensitive genotype mean separation than the other indicators. This characteristic, however, requires multiple freezing temperatures (four in our study), making it less suitable for evaluating large genotype numbers than the frost tolerance assessment based on one optimal freezing temperature (i.e., the one that maximizes the genotype variation for plant mortality). The optimal freezing temperature differed for the two species according to our results, being about −13°C for pea and −11°C for white lupin. When used for evaluation at an optimal freezing temperature, our platform could accommodate up to 216 genotypes in each of several evaluation cycles (each cycle acting as a replicate), using experimental units (replicates) of 10 plants each as in this study (or 144 genotypes, using experimental units of 15 plants). Our results suggest that the biomass injury score may concur to the frost tolerance evaluation along with plant mortality or act as the only frost tolerance indicator in case the platform included more genotypes per evaluation cycle with less plants per experimental unit (e.g., 432 genotypes with five plants per replicate), a situation that makes the estimation of plant mortality less reliable. A similar score was adopted by Beji et al. (2020) in a pea experiment including four plants per replicate, and is frequently adopted in other grain legumes under similar circumstances (Arbaoui and Link, 2008). Work by Humplík et al. (2015) suggests that the biomass injury assessment of the individual plants could be automated by image analysis, albeit hardly with large time savings and with a need for placing plants into individual pots.

The optimal freezing temperature for pea plant mortality at −13°C contrasts with earlier results by Auld et al. (1983), Swensen and Murray (1983), Murray et al. (1988), Cousin et al. (1993), and Homer et al. (2016), which suggested an optimal temperature in the range of −7°C to −9°C. The contrast is even greater when considering that most of these studies adopted a longer hardening period than our study. The improved frost tolerance of the current, recently bred germplasm sample (breeding lines or commercial cultivars), along with possible differences in the evaluation protocols, may partly account for the currently lower optimal freezing temperature. For example, differences in substrate type and drainage may affect plant mortality (Russell et al., 1978). Irrigation during the hardening period (as contemplated in some earlier studies) could increase ice formation and cause mechanical damage to the roots. Our slower thawing (1°C/h) relative to some early studies could be less damaging to plants (Gusta and Fowler, 1977). Other possibly different factors may include the plant growth and development stage before hardening, the duration of the frost treatment, and the length of the regrowth period. Although the frost tolerance evaluation of pea germplasm collections at −8°C is quite frequent (Wery et al., 1994; Dumont et al., 2009; Beji et al., 2020), Prieur and Cousin (1978) suggested the selection of frost-tolerant pea germplasm by a set of freezing cycles ultimately achieving −12°C.

No prior assessment of genotype frost tolerance variation and optimal temperature for frost tolerance evaluation based on LT_50_ for plant mortality was available for white lupin. A study based on frost-induced leaf damage of cultivars and accessions estimated by chlorophyll fluorescence indicated an average value of −9.5°C for LT_50_, estimated as 50% of damaged leaves after a long hardening period (42 days at 8°C/2°C day/night temperature) (Hoffmann-Bahnsen and Herzog, 2001). In contrast, Papineau and Huyghe (1992) proposed to assess white lupin frost tolerance at −16°C freezing temperature after a 3-week hardening period at −4°C.

The observed good consistency between platform-based frost tolerance and field-based winter hardiness of pea and white lupin genotypes has practical importance for the exploitation of artificial screening results for these species. Correlations for pea plant mortality across field and growth chamber assessments were close to 0.7 in Homer et al. (2016) and in the range of 0.5–0.6 in Auld et al. (1983). Correlations close to 0.5 have been reported for other legume species such as faba bean (Arbaoui et al., 2008) and red clover (Zanotto et al., 2021). As anticipated, one cannot expect a very high consistency between platform-based and field-based plant mortality in grain legumes because the latter depends not only on intrinsic frost tolerance but also on frost avoidance through a delayed onset of flowering. Other factors may influence the genotype variation for field plant survival, such as greater tolerance to diseases whose attack is favored by frost damage, such as *Ascochyta* spp. for pea (Maufras, 1997), and a different susceptibility to imbibitional chilling of the germinating seed due to seed coat variation for rapidity of imbibition (Wery et al., 1994). Onset of flowering may actually explain the response of the lupin breeding line PLI-P3, which featured moderate frost tolerance according to the freezing test while belonging to the low winter hardiness class according to field observations. The high winter mortality under field conditions of this line was associated with extreme earliness of flowering in Annicchiarico et al. (2011) (where this line is coded as P3), a feature that would definitely increase its sensitivity to frost because of the early differentiation of the floral apex (Huyghe and Papineau, 1990). The currently good but not outstanding intrinsic frost tolerance of the pea landrace Champagne (**Table 2**) in spite of its reportedly extreme field-based winter survival (Prieur and Cousin, 1978; Dumont et al., 2011) may be accounted for by frost escape under field conditions via delayed flowering caused by possession of the *Hr* (high response to photoperiod) gene (Dumont et al., 2009). Indeed the *Hr* gene reportedly co-segregated with the most important quantitative trait loci (QTL) for frost tolerance (Lejeune-Hénaut et al., 2008). Anyway, the reliability of our genotype classifications for winter hardiness suffered from the limited field-based evaluation it was based upon. For example, the pea variety Dove, here classified as intermediate for winter hardiness while showing high frost tolerance according to freezing test results, exhibited moderately high frost tolerance across various cold-prone agricultural environments of France (UNIP-ITCF, 2001).

In conclusion, our results encourage the use of high-throughput phenotyping platforms such as the current one for the assessment of pea or white lupin frost tolerance aimed at plant breeding, molecular studies for detection of QTL (e.g., Beji et al., 2020) and/or definition of genome-enabled prediction models, or for investigation of physiological mechanisms regulating frost tolerance (e.g., Dumont et al., 2009).

The assessment under artificial conditions could overcome the increasing unpredictability of field-based evaluations. In addition, its focus on intrinsic frost tolerance (as implied by the evaluation of young plants that lack any differentiation of reproductive organs) facilitates the combination of cold tolerance and drought tolerance characteristics unrelated to flowering time in novel varieties featuring greater yield stability and adaptation to the increasingly variable climate conditions. Indeed our results for PLI-P3 and Champagne confirm that intrinsic frost tolerance is not necessarily related to plant mortality under field conditions for pea or white lupin genotypes, and a similar response was observed for a few faba bean genotypes (Arbaoui et al., 2008). For pea, a genomic selection model for intrinsic drought tolerance proved capable of producing material with a similar flowering time but with increased yielding ability under severe drought relative to its genetic base (Annicchiarico et al., 2020), while a similar model is awaiting exploitation for white lupin (Pecetti et al., 2023).

## Data Availability

The raw data supporting the conclusions of this article will be made available by the authors, without undue reservation.
