# Supplementary figures and images for: Frost tolerance improvement in pea and white lupin by a high-throughput phenotyping platform
Source: Front Plant Sci. 2024 Dec 20;15:1490577. doi: 10.3389/fpls.2024.1490577 (PMC11695127; doi:10.3389/fpls.2024.1490577)

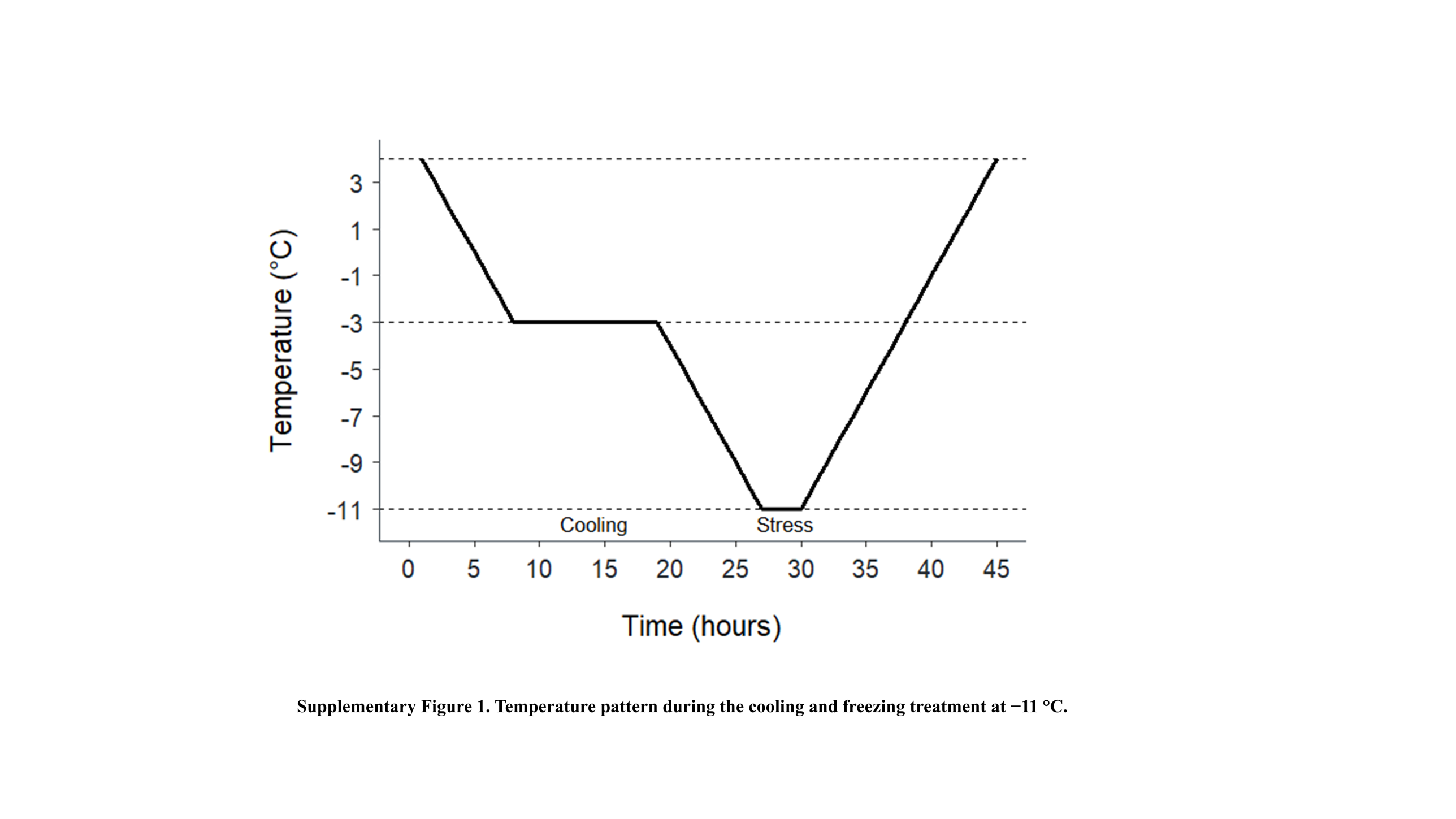

Supplement: Supplementary file 1 [file Image1.tif]
