# Supplementary material for: Frost tolerance improvement in pea and white lupin by a high-throughput phenotyping platform
Source: Front Plant Sci. 2024 Dec 20;15:1490577. doi: 10.3389/fpls.2024.1490577 (PMC11695127; doi:10.3389/fpls.2024.1490577)
Supplement: Supplementary file 2 [file Table1.docx]

**Supplementary Table 1. Experimental protocol summarizing temperature, length, light hours/day, and irrigation management of each phase.**

|  | Length | Temperature | Light hours/day | Irrigation |
| --- | --- | --- | --- | --- |
| Germination | 48 hours | 19 °C | Dark |  |
| Growth | 10 days | 22.5 °C | 12 h | Yes |
| Hardening | 15 days | 4 °C | 10 h | No |
| Cooling | 12 hours | −3 °C | Dark | No |
| Stress | 4 hours | −7 °C; −9 °C; −11 °C; −13 °C | Dark | No |
| Recovery | 6 days | 4 °C | 10 h | Yes |
| Regrowth | 15 days | 15/20 °C (night/day) | 12 h | Yes |
