# Supplementary material for: Frost tolerance improvement in pea and white lupin by a high-throughput phenotyping platform
Source: Front Plant Sci. 2024 Dec 20;15:1490577. doi: 10.3389/fpls.2024.1490577 (PMC11695127; doi:10.3389/fpls.2024.1490577)
Supplement: Supplementary file 3 [file Table2.docx]

**Supplementary Table 2.** **Correlations for LT_50_ value, plant mortality proportion at two freezing temperatures and biomass injury visual score (VS) after four freezing temperatures of 11 pea genotypes.**

| Trait | Mortality  post −11 °C | Mortality  post −13 °C | VS  post −7 °C | | VS  post −9 °C | VS  post −11 °C | VS  post −13 °C |
| --- | --- | --- | --- | --- | --- | --- | --- |
| LT_50_ | 0.81** | 0.91*** | | 0.64* | 0.75** | 0.85** | 0.92*** |
| Mortality post −11 °C | − | 0.81** | | 0.90*** | 0.86*** | 0.87*** | 0.83** |
| Mortality post −13°C | − | − | | 0.79** | 0.76** | 0.75** | 0.98*** |
| VS post −7 °C | − | − | | − | 0.89*** | 0.77** | 0.81** |
| VS post −9 °C | − | − | | − | − | 0.85*** | 0.82** |
| VS post −11°C | − | − | | − | − | − | 0.84** |

| **P* < 0.05; ***P* < 0.01, ****P* < 0.001; NS not significant (*P* >0.05) |
| --- |
