# Supplementary material for: Frost tolerance improvement in pea and white lupin by a high-throughput phenotyping platform
Source: Front Plant Sci. 2024 Dec 20;15:1490577. doi: 10.3389/fpls.2024.1490577 (PMC11695127; doi:10.3389/fpls.2024.1490577)
Supplement: Supplementary file 4 [file Table3.docx]

**Supplementary Table 3. Correlations for LT_50_ value, plant mortality at two freezing temperatures and biomass injury visual score (VS) after two freezing temperatures of 11 white lupin genotypes.**

| Trait | Mortality post −11 °C | Mortality post −13 °C | VS post −9 °C | VS post −11 °C |
| --- | --- | --- | --- | --- |
| LT_50_ | 0.94*** | 0.90*** | 0.78** | 0.91*** |
| Mortality post −11 °C | − | 0.78** | 0.76** | 0.97*** |
| Mortality post −13 °C | − | − | 0.50 NS | 0.76** |
| VS post −9 °C | − | − | − | 0.78** |

| **P* < 0.05; ***P* < 0.01, ****P* < 0.001; NS not significant (*P* >0.05) |
| --- |
